# The predictive study of the relation between elevated low-density lipoprotein cholesterol to high-density lipoprotein cholesterol ratio and mortality in peritoneal dialysis

**DOI:** 10.1186/s12944-020-01240-8

**Published:** 2020-03-21

**Authors:** Tong Lin, Xi Xia, Jing Yu, Yagui Qiu, Chunyan Yi, Jianxiong Lin, Haiping Mao, Xiao Yang, Fengxian Huang

**Affiliations:** 1 Department of Nephrology, the First Affiliated Hospital of Sun Yat-sen University, Guangzhou, 58th, Zhongshan Road II, Guangzhou, 510080 People’s Republic of China; 2 Key Laboratory of Nephrology, National Health Commission of China and Guangdong Province, Guangzhou, 510080 People’s Republic of China

**Keywords:** Low-density lipoprotein cholesterol to high-density lipoprotein cholesterol ratio (LDL-C/HDL-C), Peritoneal dialysis, Cardiovascular mortality, All-cause mortality

## Abstract

**Background:**

The low-density lipoprotein cholesterol to high-density lipoprotein cholesterol (LDL-C/HDL-C) ratio constitutes a strong risk predictor of cardiovascular events. However, the association between this ratio and cardiovascular death in peritoneal dialysis (PD) patients is uncertain. The study aimed to investigate whether a high LDL-C/HDL-C ratio could predict both cardiovascular and all-cause mortalities in patients on PD.

**Methods:**

A total of 1616 incident patients on PD included from January 1, 2006 to December 31, 2013 were followed up with until 31 December 2018 in this single-center prospective cohort study. Participants were divided into three categories according to LDL-C/HDL-C ratio tertile. The primary endpoint was cardiovascular mortality; the secondary endpoint was all-cause mortality.

**Results:**

The mean age of the study cohort was 47.5 years and the mean body mass index (BMI) was 21.6 kg/m^2^. During a median follow-up period of 47.6 months, 492 patients died, including 246 (50.0%) due to cardiovascular disease (CVD). A multivariate analysis revealed that the highest LDL-C/HDL-C ratio tertile was significantly associated with increased CVD mortality [hazard ratio (HR): 1.69, 95% CI: 1.24–2.29; *P =* 0.001] and all-cause mortality (HR: 1.46, 95% CI: 1.18–1.81; *P =* 0.001) relative to the lowest tertile. After adjusting for covariates, the HRs of cardiovascular and all-cause mortalities were 1.84 (95% CI: 1.25–2.71; *P =* 0.002) and 1.35 (95% CI: 1.03–1.77; *P =* 0.032). Subgroup analysis showed that the risk of CVD death rose with a higher LDL-C/HDL-C ratio among PD patients who were female, younger than 65 years old, without being malnourished (BMI ≥ 18.5 kg/m^2^ or albumin ≥35 g/L), and with a history of diabetes or CVD, respectively.

**Conclusions:**

A high LDL-C/HDL-C ratio is an independent risk factor for both cardiovascular and all-cause mortalities among PD patients.

## Introduction

Cardiovascular disease (CVD) is considered to be the most common cause of mortality among patients with chronic kidney disease (CKD) [1–3]. Accounting for nearly 50% of deaths among patients on dialysis, cardiovascular mortality in this group is 10 to 30 times higher than that in the general population [4, 5].

Hyperlipidemia, especially hypercholesterolemia, associated with the development of atherosclerosis, is often found in patients with end-stage renal disease (ESRD). Patients undergoing continuous ambulatory peritoneal dialysis (CAPD) tend to show elevated levels of total cholesterol (TC) and low-density lipoprotein cholesterol (LDL-C) and decreased levels of high-density lipoprotein cholesterol (HDL-C) [6]. Experimental studies in the general population have revealed that reductions in TC and LDL-C could limit cardiovascular events and mortality [7, 8]. Unfortunately, several epidemiological investigations and randomized clinical trials have failed to indicate lipid-lowering therapy is profitable in reducing CVD in dialysis patients despite the observation of significant reductions in LDL-C levels [9, 10]. Thus, LDL-C may not be the best predictor of cardiovascular risk in PD patients. Instead, evidence increasingly supports that the ratio of LDL-C/HDL-C might, in fact, be a novel marker of the risk of atherosclerotic CVD given it simultaneously evaluates the levels of both LDL-C and HDL-C [11–13]. In the setting of coronary plaque progression and early-stage atherosclerosis, there has been a growing attention on applying this ratio for the identification of hyperlipidemia as an alternative to traditional lipid profile analysis [14, 15]. Besides, the LDL-C/HDL-C ratio was previously found to predict the risk of reduced glomerular filtration rate in H-type hypertension, providing insights into lipoprotein-mediated renal injury [16].

However, the predictive value of the LDL-C/HDL-C ratio in understanding cardiovascular events in PD patients remains unclear. Therefore, the present cohort study was conducted to investigate the impact of LDL-C/HDL-C level in predicting cardiovascular and all-cause mortality rates among PD patients.

## Materials and methods

### Participants

All incident patients who began CAPD therapy at the First Affiliated Hospital of Sun Yat-sen University (SYSU) in China from January 1, 2006 to December 31, 2013 were recruited. The inclusion criteria were age of 18 years or older at the initiation of PD therapy and having received at least three consecutive months of CAPD therapy. Patients with malignant disease, who were transferred from chronic hemodialysis (HD) or who failed kidney transplantation, and who lack of complete lipid data were excluded. Conventional PD solutions (Dianeal 1.5, 2.5%, or 4.25% dextrose; Baxter Healthcare, Guangzhou, China), Y-sets, and twin-bag systems were applied in almost all of the CAPD cases. This study was performed in accordance with the ethical principles of the Declaration of Helsinki and was approved by the human ethics committees of SYSU. Written informed consent was obtained from all participants before enrollment.

### Study protocol

This was a single-center prospective cohort study. All patients were followed up with until death, transfer to HD, kidney transplantation, transfer to another center, or censoring on December 31, 2018. Baseline demographic [i.e., age, gender, cause of ESRD, body mass index (BMI)], major comorbidities (e.g., CVD, hypertension, diabetes mellitus), and clinical data were collected at the initiation of PD. The collected biochemical data included baseline hemoglobin, serum albumin, serum creatinine, uric acid, high-sensitive C-reactive protein (hs-CRP), TC, triglycerides, HDL-C, and LDL-C were measured in the center laboratory of the First Affiliated Hospital of SYSU. The dialysis dose (Kt/V) was calculated according to 24-h dialysate collection using the PD Adequest software (Baxter Healthcare, Guangzhou, China). The estimated glomerular filtration rate (eGFR) was calculated as the mean of urea and creatinine clearance, calculated from 24-h urine collections and adjusted for a body surface area of 1.73 m^2^. The International Society for Peritoneal Dialysis guidelines were adopted to evaluate and manage any dyslipidemia. Medication usage data were derived from prescriptions. Lipid-lowing drugs included statins and fibrates. Study participants were asked to visit our center quarterly for a comprehensive medical evaluation and were also interviewed by telephone monthly by trained nurses to assess general health status and concomitant medication usage. These quarterly visits and monthly telephone contacts were performed for clinical purposes rather than specifically for this study.

The primary outcome of interest was cardiovascular mortality, while all-cause mortality was considered as the secondary outcome of interest. Cardiovascular death was defined as death caused by acute myocardial infarction, atherosclerotic heart disease, cardiac arrhythmia, cardiomyopathy, cardiac arrest, congestive heart failure, cerebrovascular accident, ischemic brain damage, anoxic encephalopathy, or peripheral vascular disease events as determined by the PD nurses and professors. Patients with the following cardiovascular events were considered to have a history of CVD: angina pectoris, myocardial infarction, angioplasty, coronary artery bypass, heart failure, or stroke.

### Statistical analysis

The LDL-C/HDL-C ratio was calculated by dividing the LDL-C value by the HDL-C value (expressed in milligrams per deciliter). Non–HDL-C was calculated by the subtraction of HDL-C from the TC level and analyzed as a continuous variable. Participants were divided into the following three groups according to LDL-C/HDL-C level tertile: T1 (< 2.04), T2 (2.04–2.74), and T3 (≥2.74). Baseline patient characteristics were compiled for each group. Continuous variables are expressed as means and the standard deviations or medians with interquartile ranges, while categorical variables are expressed as frequencies with percentages. Chi-squared, one-way analysis of variance, or Kruskal–Wallis tests were employed to examine differences among groups. Furthermore, logistic analysis was conducted to identify risk factors for high LDL-C/HDL-C ratio. Multivariate logistic regression model was built after using univariate model to eliminate variables that failed to have significant effect (*P* > 0.1). Survival was analyzed using the Kaplan–Meier method, and the distributions of survival were compared using a log-rank test. Cox’s proportional-hazards regression was employed to assess the association between LDL-C/HDLC-C ratio and mortality rate with or without adjustment for covariates. Covariates with *P* <  0.1 in univariate models or for importance of clinical concern were selected for multivariate Cox regression modeling. Since the LDL-C/HDL-C ratio was calculated from the original lipid data, a high correlation with the original lipid profile may lead to excess collinearity and potential spurious results, so lipid profiles were not included in the multivariate Cox regression model. The effects of different lipid levels were analyzed after stratification according to lipid level in the subgroup analyses. The results are presented as hazard ratios (HRs) and 95% confidence intervals (95% CIs). Furthermore, forest plots were constructed to identify whether adjusted HRs of increased LDL-C/HDL-C ratios differed significantly between subgroups according to baseline characteristics, lipid level, or statin treatment. Statistical significance was defined as *P* <  0.05 using two-tailed tests. Statistical analyses were performed using the Statistical Package for the Social Sciences version 23.0 for Mac software program (IBM Corp., Armonk, NY, USA).

## Results

### Patient characteristics

**Fig. 1:**
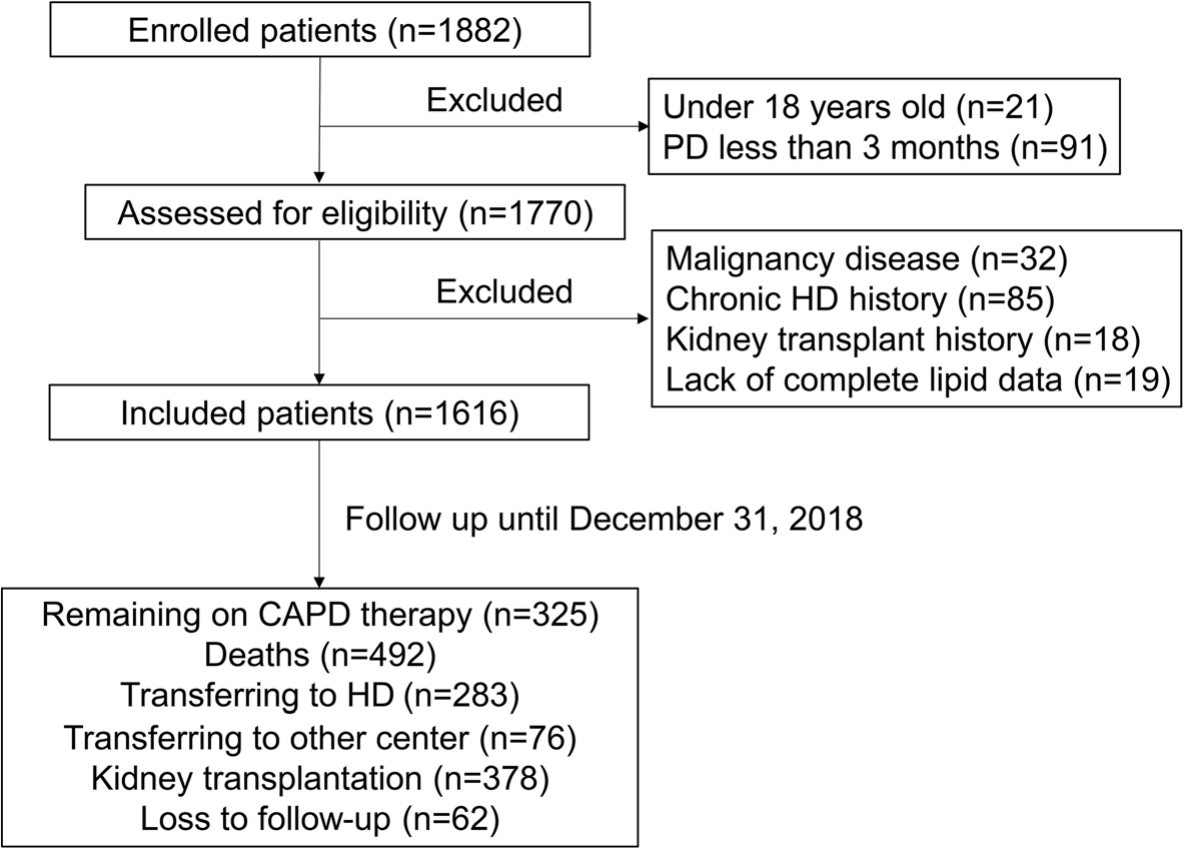
Flow chart of the participants in the study cohort. PD, peritoneal dialysis; HD, hemodialysis

A total of 1616 incident PD patients were eligible for inclusion in the final analysis (Fig. 1). Among these, 59.8% of the patients were male, the mean age was 47.5 ± 15.2 years old, and the mean BMI was 21.6 ± 3.1 kg/m at the initiation of dialysis. The primary cause of ESRD was chronic glomerulonephritis (52.8%), followed by diabetes nephropathy (22.8%) and hypertension (7.2%). The mean baseline LDL-C/HDL-C ratio was 2.5 ± 1.0 as the serum mean LDL-C was 113.3 ± 38.8 mg/dL and mean HDL-C was 47.6 ± 14.9 mg/dL. The baseline characteristics of patients according to LDL-C/HDL-C ratio tertile are summarized in Table 1. Patients with higher LDL-C/HDL-C levels were typically older and more likely to be female; exhibited a higher prevalence of diabetes mellitus; and had higher levels of BMI, hemoglobin, hs-CRP, serum uric acid, TC, triglycerides, serum LDL-C, non–HDL-C, apo B, and apo B/apo A1 ratio but lower levels of serum HDL-C and apoA1 (*P <* 0.05). There were no significant differences observed between the groups in terms of blood pressure, serum albumin, serum creatinine, eGFR, total Kt/V urea, hypertension, CVD, or statin treatment (*P* > 0.05). The multivariate logistic analysis revealed that high LDL-C/HDL-C ratio (T3) was significantly associated with female [odds ratio (OR), 1.337; 95% confidence interval (CI), 1.016–1.761; *P* = 0.038], higher BMI (OR, 1.190; 95% CI,1.135–1.248; *P* <  0.001), higher hsCRP (OR, 1.086; 95% CI,1.051–1.123; *P* < 0.001), higher TC (OR, 1.029; 95% CI,1.025–1.034; P < 0.001), and TG level (OR,1.003; 95% CI, 1.001–1.005; *P* = 0.014) (Supplementary Table 1).

**Table 1:** Baseline patient characteristics according to LDL-C/HDL-C tertile

Characteristic	Total (***n*** = 1616)	LDL-C/HDL-C Ratio Tertile			***P***-value
		T1 (< 2.04) (*n* = 539)	T2 (2.04–2.74) (*n* = 538)	T3 (≥2.74) (*n* = 539)	
Age (years)	47.5 ± 15.2	46.0 ± 15.1	47.4 ± 15.3	49.2 ± 15.1	**0.003**
Male gender, n (%)	966 (59.8)	239 (44.3)	215 (40.0)	196 (36.4)	**0.028**
BMI (kg/m^2^)	21.6 ± 3.1	20.8 ± 2.9	21.6 ± 3.2	22.4 ± 3.1	**< 0.001**
Systolic pressure (mmHg)	136.5 ± 19.8	136.4 ± 19.8	136.0 ± 19.8	137.2 ± 19.7	0.575
Diastolic pressure (mmHg)	84.7 ± 14.4	84.8 ± 15.0	85.3 ± 13.7	84.2 ± 14.5	0.468
Diabetes, n (%)	415 (25.7)	122 (22.6)	125 (23.2)	168 (31.2)	**0.002**
CVD n (%)	596 (36.9)	202 (37.5)	192 (35.7)	202 (37.5)	0.781
Hypertension, n (%)	1439 (89.0)	484 (89.8)	470 (87.4)	485 (90.0)	0.307
Total cholesterol (mg/dL)	196.5 ± 51.4	174.5 ± 43.7	194.4 ± 43.3	220.4 ± 55.8	**< 0.001**
Triglycerides (mg/dL)	124.8 (89.4–177.0)	95.6 (70.3–137.2)	127.4 (94.7–171.7)	154.9 (114.2–210.2)	**< 0.001**
HDL-C (mg/dL)	47.6 ± 14.9	56.5 ± 16.7	46.9 ± 11.8	39.5 ± 10.3	**< 0.001**
LDL-C (mg/dL)	113.3 ± 38.8	88.6 ± 26.7	111.3 ± 27.9	140.1 ± 41.0	**< 0.001**
LDL-C/HDL-C	2.5 ± 1.0	1.6 ± 0.3	2.4 ± 0.2	3.6 ± 0.9	**< 0.001**
Non-HDL-C (mg/dL)	148.8 ± 48.1	118.1 ± 34.4	147.5 ± 35.5	181.0 ± 50.2	**< 0.001**
Hemoglobin (g/L)	105.0 ± 21.2	103.1 ± 21.7	106.8 ± 21.2	105.1 ± 20.5	**0.014**
Serum albumin (g/L)	37.3 ± 5.2	37.2 ± 5.0	37.6 ± 5.0	37.0 ± 5.4	0.128
Creatinine (mg/dL)	8.7 ± 3.2	8.7 ± 3.3	8.6 ± 3.1	8.8 ± 3.4	0.373
Uric acid (μmol/L)	417.2 ± 91.9	408.4 ± 94.2	415.1 ± 85.8	428.0 ± 94.4	**0.002**
Hs-CRP (mg/L)	1.8 (0.6–5.7)	1.2 (0.4–3.6)	1.5 (0.7–5.7)	2.9 (1.0–8.1)	**< 0.001**
eGFR (mL/min/1.73 m^2^)	6.9 ± 3.1	6.8 ± 3.0	7.0 ± 3.4	6.7 ± 3.0	0.431
Kt/V	2.5 ± 0.7	2.5 ± 0.7	2.5 ± 0.6	2.4 ± 0.7	0.750
Statins n (%)	236 (14.6)	91 (16.9)	71 (13.2)	74 (13.7)	0.180

*BMI* Body Mass Index, *CVD* Cardiovascular disease, *HDL-C* high-density lipoprotein cholesterol, *LDL-C* low-density lipoprotein cholesterol, *hs-CRP* High-sensitive C-reactive protein, *eGFR* Estimated glomerular filtration rate

*P* < 0.05 is considered to be statistically significant

### Association between LDL-C/HDL-C ratio and CVD mortality or all-cause mortality

**Fig. 2:**
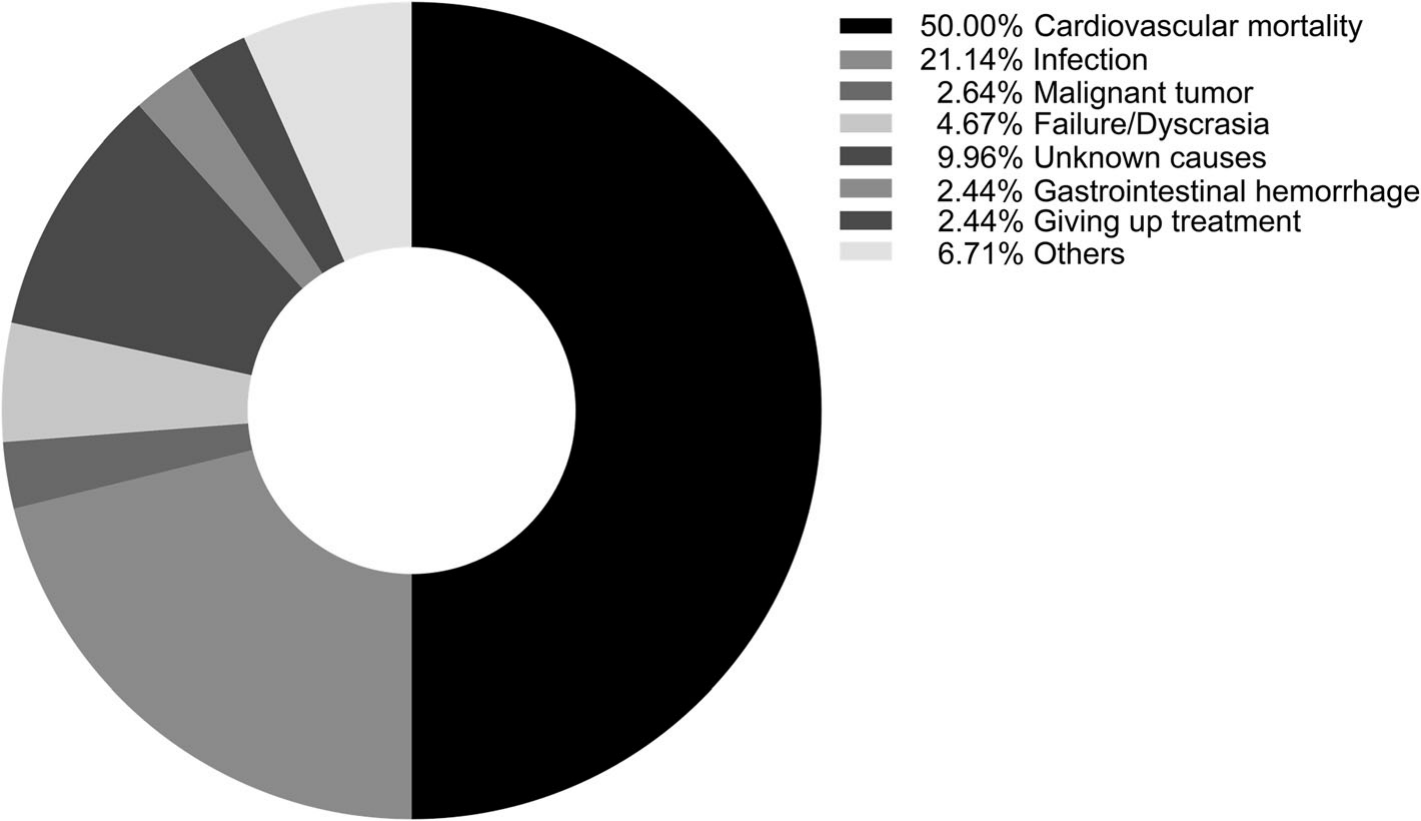
Causes of death in the study cohort

The median follow-up period was 47.6 months (range: 24.2–82.7 months). By the end of this study, 492 (30.4%) patients had died, 378 (23.4%) patients had undergone kidney transplantation, 283 (17.5%) patients were transferred to the HD, 76 (4.7%) patients were transferred to another PD center, and 62 (3.8%) patients were lost to follow-up (Fig. 1). Among the 492 deaths, 246 deaths (50%) were caused by cardiovascular events, while the other reasons are presented in Fig. 2. A total of 154 deaths (70 due to CVD) occurred in the lowest tertile group, whereas 145 (74 due to CVD) and 193 (102 due to CVD) deaths occurred in the middle and highest tertiles, respectively (Table 2).

**Table 2:** The association of LDL-C/HDL-C ratio with CVD and all-cause mortalities in the study cohort

	LDL-C/HDL-C Tertile					HR per 0.5 increased	
	T1	T2		T3			
CVD mortality							
Event rate, %	13.0	13.8		18.9		15.2	
Events/total	70/539	74/538		102/539		246/1616	
HR (95% CI), P-value							
Unadjusted	1 [reference]	1.113 (0.802–1.543)	0.522	**1.689 (1.244–2.291)**	**0.001**	1.112 (1.056–1.171)	< 0.001
Model 1	1 [reference]	1.079 (0.777–1.496)	0.651	1.471 (1.084–1.998)	0.013	1.087 (1.027–1.150)	0.004
Model 2	1 [reference]	1.121 (0.805–1.561)	0.498	1.476 (1.079–2.018)	0.015	1.079 (1.018–1.144)	0.010
Model 3	1 [reference]	1.338 (0.901–1.987)	0.149	1.732 (1.191–2.518)	0.004	1.110 (1.037–1.188)	0.003
Model 4	1 [reference]	1.423 (0.954–2.122)	0.084	**1.880 (1.280–2.760)**	**0.001**	1.123 (1.049–1.202)	0.001
All-cause mortality							
Event rate, %	28.6	27.0		35.8		30.4	
Events/total	154/539	145/538		193/539		492/1616	
HR (95% CI), *P*-value							
Unadjusted	1 [reference]	0.994 (0.792–1.247)	0.958	**1.462 (1.182–1.808)**	**0.001**	1.099 (1.058–1.142)	< 0.001
Model 1	1 [reference]	0.952 (0.759–1.195)	0.673	1.255 (1.014–1.552)	0.037	1.070 (1.027–1.115)	0.001
Model 2	1 [reference]	0.984 (0.782–1.237)	0.887	1.248 (1.004–1.553)	0.046	1.065 (1.020–1.110)	0.004
Model 3	1 [reference]	1.077 (0.817–1.420)	0.597	1.318 (1.010–1.721)	0.042	1.073 (1.018–1.131)	0.009
Model 4	1 [reference]	1.094 (0.828–1.446)	0.529	**1.345 (1.026–1.765)**	**0.032**	1.077 (1.021–1.136)	0.006

Model 1: Adjusted for age and gender

Model 2: Adjusted for model 1 covariates and diabetes, history of cardiovascular events, BMI, and systolic blood pressure

Model 3: Adjusted for model 2 covariates and hemoglobin, serum albumin, uric acid, serum Cr, hs-CRP, Kt/V, and eGFR

Model 4: Adjusted for model 3 covariates and statin use

*P* < 0.05 is considered to be statistically significant

**Fig. 3:**
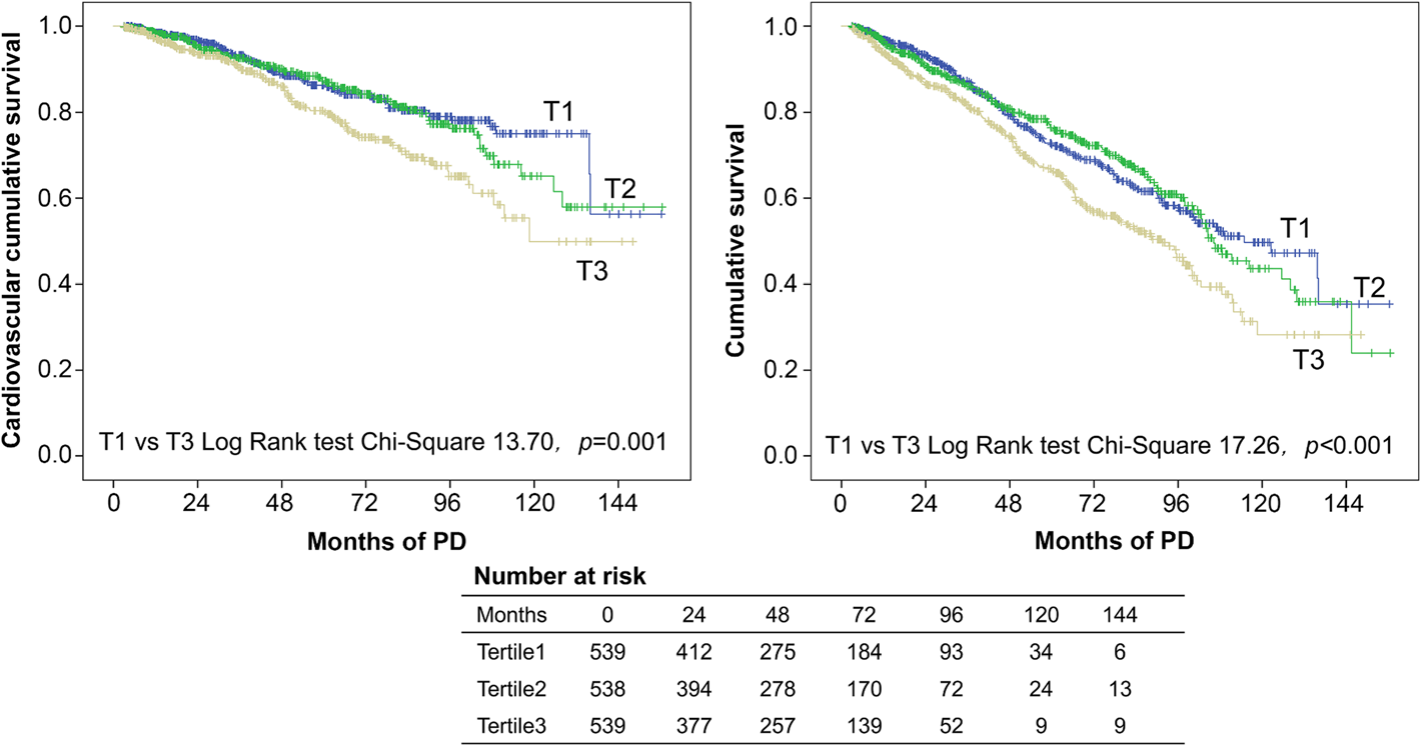
Kaplan–Meier curves of PD patients with different levels of LDL-C/HDL-C ratio cardiovascular mortality (A) and all-cause mortality (B).

The Kaplan–Meier survival curves for CVD and all-cause mortality rates according to the different levels of LDL-C/HDL-C ratio are illustrated in Fig. 3. The CVD and all-cause mortality rates were found to vary significantly between the three groups (*P =* 0.001 and *P <* 0.001, log-rank test). Patients in the T3 group exhibited the lowest cardiovascular and overall survival rates.

To investigate potential predictors of CVD and all-cause mortality, a univariate Cox proportional-hazards analysis was carried out (Supplementary Table 2). This univariate analysis revealed that the following characteristics were significantly associated with both higher CVD and all-cause mortality rates: older age; presence of DM and CVD; use of statins, higher BMI and levels of TC, TG, non-HDL, LDL-C/HDL-C ratio, eGFR, and total Kt/V; and lower hemoglobin, serum albumin, and serum creatinine levels. Of note, LDL-C/HDL-C ratio was the most relevant among these characteristics (HR for CV mortality: 1.24, 95% CI: 1.11–1.37; *P <* 0.001 and HR for all-cause mortality: 1.21, 95% CI: 1.12–1.30; P *<* 0.001). As shown in Table 2, a multivariate Cox proportional hazard analysis indicated that the high LDL-C/HDL-C ratio was an independent predictor of CVD mortality and all-cause mortality. The highest LDL-C/HDL-C tertile was associated with significantly higher CVD mortality (HR: 1.69, 95% CI: 1.24–2.29; *P* = 0.001) and all-cause mortality (HR = 1.46, 95% CI: 1.18–1.81; *P* = 0.001) relative to the lowest tertile. After adjustment for various covariates (model 4), the HRs of cardiovascular and all-cause mortalities were 1.84 (95% CI: 1.25–2.71; *P =* 0.002) and 1.35 (95% CI: 1.03–1.77; *P =* 0.032), respectively. Similar results were obtained when the ratio was examined as a continuous variable; the HRs for CVD and all-cause mortality rates associated with a 0.5 increase in the LDL-C/HDL-C ratio were 1.12 (95% CI: 1.05–1.20; *P* = 0.001) and 1.08 (95% CI: 1.02–1.14; *P* = 0.006), respectively.

Subgroup analyses were carried out to assess further associations between LDL-C/HDL-C ratio and the risk of cardiovascular mortality in different clinically relevant subgroups. The results according to such are shown in Fig. 3. The risk of CVD death statistically significantly increased with the rise in LDL-C/HDL-C ratio in female PD patients, patients younger than 65 years old, patients with BMI ≥ 18.5 kg/m^2^ or albumin ≥35 g/L, and patients with a history of diabetes or CVD. Among subgroups stratified by lipid profile, patients with HDL-C ≥ 35 mg/dL, TC ≥ 230 mg/dL, and TG ≥ 150 mg/dL had HRs of 1.88 (95% CI: 1.33–2.67; *P* < 0.001), 3.28 (95% CI: 1.27–8.51; *P* = 0.014) and 1.81 (95% CI: 1.00–3.26; *P* = 0.049), respectively.

## Discussion

Dyslipidemia is a well-recognized risk factor for CVD in the general population, but its relationship in the CKD population is not clear. In the last few decades, numerous clinical and genetic studies unequivocally have indicated that LDL-C is causally relevant to atherosclerotic CVD [17]. The Cholesterol Treatment Trialists’ collaborative meta-analysis of individual participant data from 26 randomized trials showed that lowering serum LDL-C with a statin therapy effectively reduced the risk of ischemic stroke, myocardial infarction, coronary revascularization procedures, and coronary death by approximately one-fifth per 1 mmol/L LDL-C reduction in a large proportion of people [18]. Elsewhere, the SHARP trial revealed that reduced serum LDL-C levels were associated with a lower risk of CVD in patients with CKD, although there was no statistically significant benefit seen in the primary endpoint in patients already receiving hemodialysis [19]. Similar results were observed in two previous large randomized trials (AURORA and 4D) considering statin usage and cardiovascular events in patients on dialysis [20, 21]. Hence, more considerations and confounders should be taken into account to assess the real value of LDL-C in dyslipidemia at the stage of ESRD.

HDL-C, generally considered to be “good” cholesterol, has various positive effects such as reverse cholesterol transportation from plaque tissue as well as anti-oxidation, anti-apoptosis and anti-inflammatory effects [22]. Low levels of HDL-C have long been associated with a high risk of CVD [23, 24]. However, new insights from the Framingham Offspring Study indicated low and high HDL-C phenotypes are not uniformly predictive of CVD risk. TG and LDL-C represent important regulators of incident CVD risk at both ends of the HDL-C spectrum [25].

Taking these facts into consideration together, a lipid ratio similar to LDL-C/HDL-C, TG/HDL-C, has been put forth as a better predictor or treatment targets for CVD. In addition, with impaired renal function and reduced clearance, lipid abnormalities also differ in different kidney diseases. PD patients are more likely to present with elevated levels of TC, TG, and LDL-C and lower levels of HDL-C [6, 26]. A previous study from our center proved that an increased serum TG/HDL-C ratio was related with higher risks of CVD all-cause mortalities among PD patients [27]. Several studies have supported the potential of LDL-C/HDL-C in predicting atherosclerotic cardiovascular events as it essentially summarizes and combines information from the levels of LDL-C and HDL-C [11, 28, 29]. Enomoto et al. reported that an increased LDL-C/HDL-C ratio was not only a marker of atherosclerosis but might also play a causal role in the pathogenesis of human IMT progression [30]. Hong et al. reported that an elevated LDL-C/HDL-C ratio was independently correlated with diabetes in a Chinese hypertensive population [31]. In the present study, our results suggest that PD patients with high LDL-C/HDL-C ratios were at a high risk of both cardiovascular and all-cause mortality, even after adjusting for several potential confounders.

**Fig. 4:**
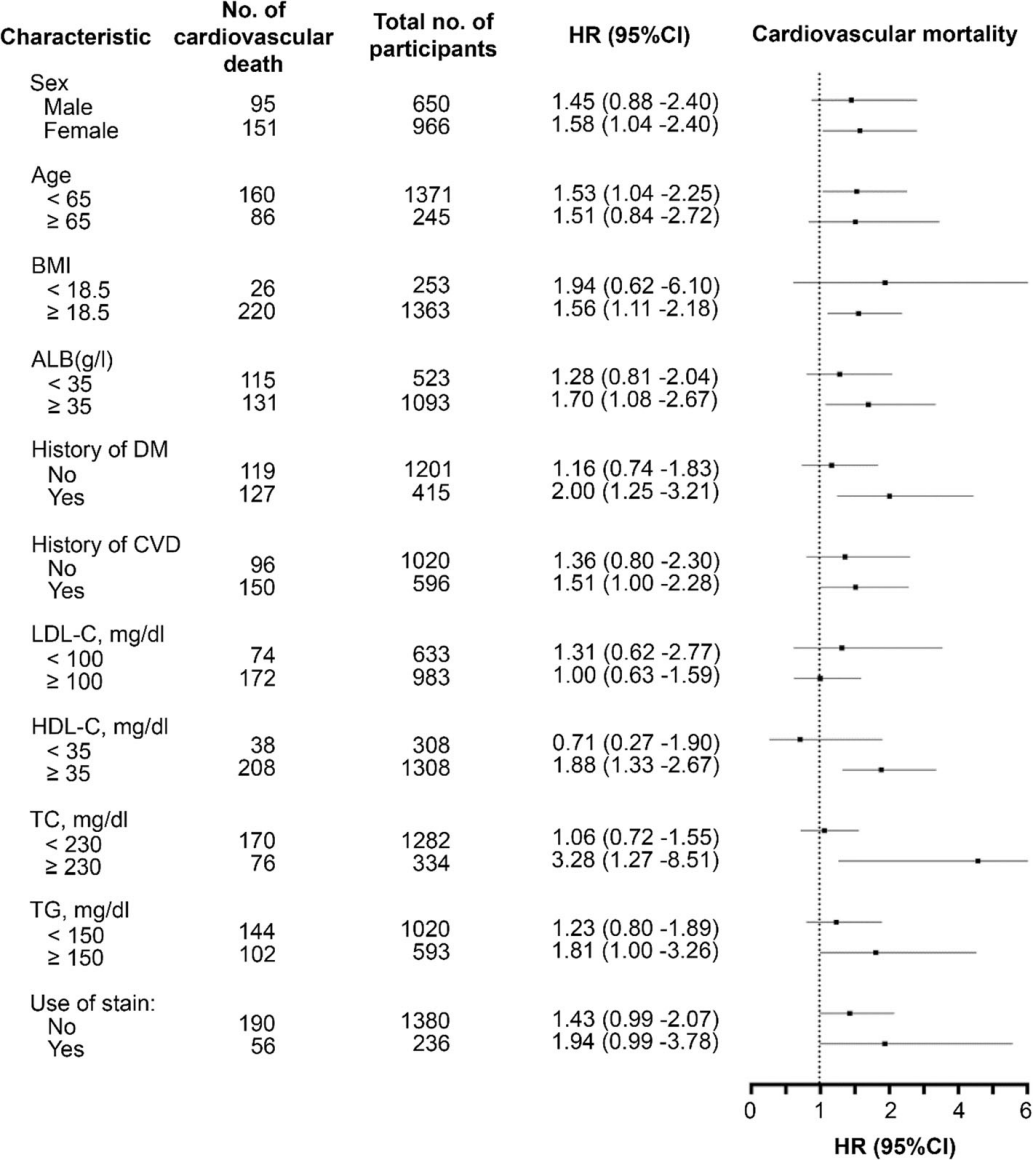
Association between LDL-C/HDL-C ratio and the risk of cardiovascular mortality stratified by patient baseline characteristics

In addition, subgroup analysis explored whether the predictive power of LDL-C/HDL-C ratio differed by gender, age, nutritional status, and comorbidities (Fig. 4). We found that, in female PD patients, patients younger than 65 years old, patients with BMI ≥ 18.5 kg/m^2^ or albumin ≥35 g/L, and patients with a history of diabetes or cardiovascular diseases, the ratio has statistical significance. It is well-known that age, diabetes mellitus, CVD, and malnutrition are strong risk factors for cardiovascular mortality. Patients with diabetes often have the lipid triad (high LDL-C, low HDL-C and high triglycerides) due to insulin deficiency or insulin resistance. These lipid abnormalities are the main cause of atherosclerosis [32]. In a study of incident PD patients, Sung et al. showed female patients with diabetes on PD are more susceptible to developing CVD than males [4]. Besides, as seen in previous NHANES surveys, women aged 30 to 49 years had lower mean LDL-C levels than men of the same age range, while women aged 70 years or older presented the opposite case. With respect to serum HDL-C levels, the levels were consistently higher in women than in men [33]. All of these lipid differences by gender and sex might partially explain the current subgroup findings. The present study also showed high LDL-C/HDL-C ratio level was associated with a higher mortality risk in patients with high HDL-C (≥35 mg/dL), high TC (≥230 mg/dL), and high TG (≥150 mg/dL) levels. The most rational explanation here might be the role of malnutrition in the relationship between mortality and cholesterol levels. Previous studies have found that an elevated risk of death is highly correlated with both low and high serum cholesterol levels, suggesting that low cholesterol levels act as an alternative marker of malnutrition with a “U-shaped” relationship between cholesterol levels and mortality [34]. For this reason, in subgroups with lower cholesterol levels, the effectiveness of the LDL-C/HDL-C ratio predicting CVD mortality was offset. Furthermore, we found that higher LDL-C/HDL-C ratio was related to higher CV mortality risk in diabetic patients with high level of hsCRP, but not in those with low level of hsCRP (Supplementary Table 3.), which is similar to previous study that high level of hsCRP was associated with the risk of cardiovascular events in patients with type 2 diabetes, and the risk would be 2-fold higher when both hsCRP and LDL-C were elevated [35]. These results implied incorporating both hsCRP and lipid parameters could provide additional stratification of cardiovascular risk in patients with diabetes. In other words, our results filled in the gap in the prediction of LDL-C/HDL-C ratio in PD patients.

Of note, there were several limitations to this study. First, the lack of biochemical data available during the follow-up period is a major limitation. Therefore, longitudinal studies are warranted to explore whether the association between LDL-C/HDL-C ratio and mortality persists or varies over time. Second, some factors that might increase the mortality rate were not adjusted for in this study, such as smoking, physical activity, dialysis insufficiency, and fluid overload. Third, due to the region and size of the cohort population, the conclusions of this study cannot be generalized to overall or other populations. Hence, further prospective cohort studies involving multiple centers and larger sample sizes need to be completed to verify the association between LDL-C/HDL-C ratio and mortality in PD patients.

## Conclusions

This is the first study in PD patients to assess the association between LDL-C/HDL-C ratio and cardiovascular and all-cause mortalities. The results remained robust despite adjustments for multiple risk factors and potential confounders. Furthermore, Subgroup analysis revealed that the risk of CVD death is statistically significantly increased with the rise in LDL-C/HDL-C ratio among female PD patients, patients younger than 65 years old, patients with BMI ≥ 18.5 kg/m^2^ or albumin ≥35 g/L, patients with a history of diabetes or cardiovascular diseases and also patients with high HDL-C (≥ 35 mg/dL), high TC (≥ 230 mg/dL), and high TG (≥150 mg/dL) levels, respectively, which suggesting a possible potential value for LDL-C/HDL-C ratio in risk prediction beyond traditional lipid profiles.

This study provides emerging evidence supporting the use of non-traditional lipid parameters in predicting cardiovascular and all-cause mortalities. More studies of large numbers of patients or meta-analyses of lipid-lowering trials for dialysis patients are warranted to determine the clinical significance of LDL/HDL-cholesterol ratio and to provide information for the development of clinical guidelines.

## Supplementary information

**Additional file 1 Supplementary Table 1**. Factors associated with high LDL-C/HDL-C ratio in Logistic regression analysis. **Supplementary Table 2**. Univariate Cox proportional-hazards analysis showing predictors of CVD and all-cause mortalities in the study cohort. **Supplementary Table 3**. The Association between LDL-C/HDL-C ratio and mortality risk by different levels of hsCRP in diabetic patients.

## Data Availability

All data generated or analyzed during this study are included in this published article.

## Abbreviations

LDL-C/HDL-C: Low-density lipoprotein cholesterol to high-density lipoprotein cholesterol ratio
PD: Peritoneal dialysis
BMI: Body mass index
CVD: Cardiovascular disease
HR: Hazard Ratio
CKD: Chronic kidney disease
ESRD: End-stage renal disease
CAPD: Continuous ambulatory peritoneal dialysis
TC: Total cholesterol
LDL-C: Low-density lipoprotein cholesterol
HDL-C: High-density lipoprotein cholesterol
SYSU: Sun Yat-sen university
HD: Hemodialysis
hs-CRP: High-sensitive C-reactive protein
eGFR: Estimated glomerular filtration rate
