# Supplementary material for: The predictive study of the relation between elevated low-density lipoprotein cholesterol to high-density lipoprotein cholesterol ratio and mortality in peritoneal dialysis
Source: Lipids Health Dis. 2020 Mar 21;19:51. doi: 10.1186/s12944-020-01240-8 (PMC7085176; doi:10.1186/s12944-020-01240-8)
Supplement: Supplementary file 1 — Additional file 1 Supplementary Table 1. Factors associated with high LDL-C/HDL-C ratio in Logistic regression analysis. Supplementary Table 2. Univariate Cox proportional-hazards analysis showing predictors of CVD and all-cause mortalities in the study cohort. Supplementary Table 3. The Association between LDL-C/HDL-C ratio and mortality risk by different levels of hsCRP in diabetic patients. [file 12944_2020_1240_MOESM1_ESM.docx]

**Supplementary Table 1. Factors associated with high LDL-C/HDL-C ratio in Logistic regression analysis**

| Variable | Univariate model | | Multivariate model | |
| --- | --- | --- | --- | --- |
|  | *OR* (95%CI) | *P* value | *OR* (95%CI) | *P* value |
| Age (years) | 1.014(1.006-1.022) | **0.001** | - | - |
| Male gender, n (%) | 1.394(1.092-1.780) | **0.008** | 1.337(1.016-1.761) | **0.038** |
| Body Mass Index (kg/m^2^) | 1.199(1.149-1.252) | **＜0.001** | 1.190(1.135-1.248) | **＜0.001** |
| Systolic pressure (mmHg) | 1.002(0.996-1.008) | 0.488 | - | - |
| Diastolic pressure (mmHg) | 0.997(0.989-1.005) | 0.524 | - | - |
| Diabetes, n (%) | 1.548(1.179-2.031) | **0.002** | - | - |
| Cardiovascular Disease, n (%) | 1.000(0.781-1.280) | 1.000 | - | - |
| Hypertension, n (%) | 1.021(0.687-1.517) | 0.920 | - | - |
| Total cholesterol (mg/dL) | 1.021(1.018-1.024) | **＜0.001** | 1.029(1.025-1.034) | **＜0.001** |
| Triglycerides (mg/dL) | 1.005(1.004-1.007) | **＜0.001** | 1.003(1.001-1.005) | **0.014** |
| Hemoglobin (g/L) | 1.004(0.999-1.010) | 0.120 | - | - |
| Serum albumin (g/L) | 0.993(0.970-1.016) | 0.541 | - | - |
| Creatinine (mg/dL) | 1.000(1.000-1.001) | 0.390 | - | - |
| Uric acid (μmol/L) | 1.002(1.001-1.004) | **0.001** | - | - |
| Hs-CRP (mg/L) | 1.105(1.07-1.141) | **＜0.001** | 1.086(1.051-1.123) | **＜0.001** |
| eGFR (mL/min/1.73 m^2^) | 0.986(0.947-1.027) | 0.493 | - | - |
| Kt/V | 0.937(0.776-1.130) | 0.495 | - | - |
| Statins n (%) | 0.783(0.561-1.093) | 0.151 | - | - |

hs-CRP, high-sensitive C-reactive protein; eGFR, estimated glomerular ﬁltration rate.

P < 0.05 is considered to be statistically signiﬁcant.

**Supplementary Table 2. Univariate Cox proportional-hazards analysis showing predictors of CVD and all-cause mortalities in the study cohort**

|  | **CVD Mortality** | | |  | **All-Cause Mortality** | | |
| --- | --- | --- | --- | --- | --- | --- | --- |
|  | Hazard ratio | 95% CI | P-value |  | Hazard ratio | 95% CI | P-value |
| Gender, male | 0.821 | 0.635–1.061 | 0.132 |  | 1.022 | 0.855–1.222 | 0.809 |
| Age (years) | 1.062 | 1.052–1.072 | < 0.001 |  | 1.064 | 1.057–1.071 | < 0.001 |
| Body Mass Index (kg/m^2^) | 1.093 | 1.051–1.136 | < 0.001 |  | 1.063 | 1.034–1.093 | < 0.001 |
| History of CVD, yes | 3.009 | 2.329–3.889 | < 0.001 |  | 2.262 | 1.894–2.701 | < 0.001 |
| History of hypertension, yes | 0.870 | 0.597–1.269 | 0.471 |  | 1.015 | 0.764–1.347 | 0.920 |
| History of DM, yes | 3.907 | 3.036–5.027 | < 0.001 |  | 3.680 | 3.079–4.399 | < 0.001 |
| Total cholesterol (mg/dL) | 1.004 | 1.002–1.007 | < 0.001 |  | 1.002 | 1.001–1.004 | 0.009 |
| Triglycerides (mg/dL) | 1.001 | 1.000–1.002 | 0.005 |  | 1.001 | 1.001–1.002 | < 0.001 |
| LDL-C (mg/dL) | 1.005 | 1.002–1.008 | 0.001 |  | 1.002 | 1.000–1.004 | 0.057 |
| HDL-C (mg/dL) | 0.992 | 0.984–1.001 | 0.086 |  | 0.989 | 0.983–0.995 | 0.001 |
| **LDL-C/HDL-C** | **1.237** | **1.115–1.372** | **< 0.001** |  | **1.208** | **1.120–1.304** | **< 0.001** |
| Non–HDL-C (mg/dL) | 1.006 | 1.003–1.008 | < 0.001 |  | 1.004 | 1.002–1.005 | < 0.001 |
| Hemoglobin (g/L) | 0.991 | 0.985–0.997 | 0.002 |  | 0.989 | 0.985–0.994 | < 0.001 |
| Serum albumin (g/L) | 0.904 | 0.883–0.926 | < 0.001 |  | 0.905 | 0.89–0.920 | < 0.001 |
| Creatinine (mg/dL) | 0.899 | 0.858–0.943 | < 0.001 |  | 0.887 | 0.857–0.918 | < 0.001 |
| Uric acid (μmol/l) | 0.998 | 0.997–1.000 | 0.015 |  | 0.999 | 0.998–1.000 | 0.025 |
| hs-CRP (mg/L) | 1.029 | 1.017–1.041 | < 0.001 |  | 1.029 | 1.021–1.038 | < 0.001 |
| eGFR (ml/min/1.73m^2^) | 1.074 | 1.041–1.108 | < 0.001 |  | 1.063 | 1.038–1.088 | < 0.001 |
| Kt/V | 0.948 | 0.774–1.161 | 0.605 |  | 1.021 | 0.892–1.169 | 0.76 |
| Stain use, yes | 2.157 | 1.599–2.909 | < 0.001 |  | 1.840 | 1.475–2.296 | < 0.001 |

CVD, cardiovascular disease; DM, diabetes mellitus; HDL-C, high-density lipoprotein cholesterol; LDL-C, low-density lipoprotein cholesterol; hs-CRP, high-sensitive C-reactive protein; eGFR, estimated glomerular ﬁltration rate.

P < 0.05 is considered to be statistically signiﬁcant.

**Supplementary Table 3.** The Association between LDL-C/HDL-C ratio and mortality risk by different levels of hsCRP in diabetic patients.

| Mortality | hsCRP ＜1.75 mg/L | | hsCRP ≥1.75 mg/L | |
| --- | --- | --- | --- | --- |
|  | HR (95%CI) | P value | HR (95%CI) | P value |
| Cardiovascular | 1.94 (0.79-4.77) | 0.149 | 3.19 (1.33-7.63) | **0.009** |
| All-cause | 1.30 (0.68-2.49) | 0.428 | 2.02 (1.21-3.34) | **0.007** |

Note: Adjusted for age and gender, history of cardiovascular events, BMI, and systolic blood pressure, hemoglobin, serum albumin, uric acid, serum Cr, Kt/V, eGFR and statin use. P < 0.05 is considered to be statistically signiﬁcant.
